# Comparing gingivitis diagnoses by bleeding on probing (BOP) exclusively versus BOP combined with visual signs using large electronic dental records

**DOI:** 10.1038/s41598-023-44307-z

**Published:** 2023-10-10

**Authors:** Jay S. Patel, Daniel Shin, Lisa Willis, Ahad Zai, Krishna Kumar, Thankam P. Thyvalikakath

**Affiliations:** 1 Division of Dental Informatics, Department of Dental Public Health and Dental Informatics, Indiana University School of Dentistry (IUSD), Indianapolis, IN USA; 2 Department of Health Services Administration and Policy, College of Public Health, Temple University, Philadelphia, PA USA; 3 Bio-Health Informatics, Indiana University School of Informatics and Computing, Indiana University-Purdue University Indianapolis (IUPUI), Indianapolis, IN USA; 4 Department of Periodontology, IUSD, Indianapolis, IN USA; 5 Center for Biomedical Informatics, Regenstrief Institute, Inc., Indianapolis, IN USA

**Keywords:** Health care, Medical research, Risk factors

## Abstract

The major significance of the 2018 gingivitis classification criteria is utilizing a simple, objective, and reliable clinical sign, bleeding on probing score (BOP%), to diagnose gingivitis. However, studies report variations in gingivitis diagnoses with the potential to under- or over-estimating disease occurrence. This study determined the agreement between gingivitis diagnoses generated using the 2018 criteria (BOP%) versus diagnoses using BOP% and other gingival visual assessments. We conducted a retrospective study of 28,908 patients' electronic dental records (EDR) from January-2009 to December-2014, at the Indiana University School of Dentistry. Computational and natural language processing (NLP) approaches were developed to diagnose gingivitis cases from BOP% and retrieve diagnoses from clinical notes. Subsequently, we determined the agreement between BOP%-generated diagnoses and clinician-recorded diagnoses. A thirty-four percent agreement was present between BOP%-generated diagnoses and clinician-recorded diagnoses for disease status (no gingivitis/gingivitis) and a 9% agreement for the disease extent (localized/generalized gingivitis). The computational program and NLP performed excellently with 99.5% and 98% f-1 measures, respectively. Sixty-six percent of patients diagnosed with gingivitis were reclassified as having healthy gingiva based on the 2018 diagnostic classification. The results indicate potential challenges with clinicians adopting the new diagnostic criterion as they transition to using the BOP% alone and not considering the visual signs of inflammation. Periodic training and calibration could facilitate clinicians' and researchers' adoption of the 2018 diagnostic system. The informatics approaches developed could be utilized to automate diagnostic findings from EDR charting and clinical notes.

## Introduction

Diagnoses and classification of the extent and severity of periodontal diseases (PD) are crucial for clinicians to determine their patients' treatment and prognoses^1,2^. It is also essential for researchers to investigate the pathogenesis and effectiveness of various treatments for PD. Experts have updated the diagnostic criteria to classify PD severity and extent based on emerging evidence on environmental and systemic risk factors' influence on PD^3–5^. The criteria have been revised four times since the first PD diagnostic classification in 1966. The most recent one was proposed in 2018 by the American Academy of Periodontology (AAP) and the European Federation of Periodontology (EFP)^6,7^. This new diagnostic classification intends to facilitate documentation of PD diagnoses in general dental practices where most periodontal diseases are diagnosed and managed. However, studies have reported gaps in implementing the 2018 classification in clinical settings^8–10^, which is expected when transitioning to a new diagnostic system. In addition, considerable variations exist among dental practitioners and dental students' use of this system that could lead to under- or over-estimation of PD^6,11^. Therefore, research to clarify "grey zones" between the new and previous diagnostic classifications is necessary to facilitate improved understanding and consistency in diagnosing periodontal cases using the 2018 classification system^9^.

A significant contribution of the 2018 diagnostic classification was the establishment of a simple and objective criterion to diagnose a gingivitis case (GC, person affected by gingivitis), which is a precursor of periodontitis^5^. This new classification uses the proportion of bleeding on probing score (BOP%) as the primary criterion to diagnose a GC because it is an objective and pragmatic clinical measure leading to fewer diagnostic errors^5,12^. According to this new definition, a patient's gingivitis status is classified into three groups: no gingivitis or healthy gingiva (BOP% < 10%), localized gingivitis (BOP% ≥ 10% and ≤ 30%), or generalized gingivitis (BOP% > 30%). This classification allows further discrimination of a patient's gingivitis status based on intact or reduced periodontium^5^. The rationale for using these criteria is because gingival inflammation may not be a disease but a variant of health and therefore, a certain amount (extent/severity) of gingival inflammation is compatible with defining a patient as periodontally healthy^5,13^. Nevertheless, this new GC definition is a significant departure from previous studies that assessed the severity of gingivitis through visual assessment, such as the gingival volume and color changes and the extent of gingival inflammation through BOP%^5^. Therefore, estimating the presence of gingival inflammation signs among patients diagnosed with healthy gingiva using the new BOP% will assist dental clinicians in using the BOP% to diagnose gingivitis despite the presence of a certain amount of gingival inflammation that is considered periodontally healthy.

Previously, studies in medicine have used the electronic health record (EHR) and administrative data to validate case definitions of various diseases such as depression,^14^ hypertension,^15,16^ diabetes, and epilepsy^16^. A systematic review^17^ reported findings from 40 studies that validated the diagnostic criteria of 47 chronic diseases. These studies demonstrated the feasibility of using EHR data to verify the case definitions of chronic diseases.

The primary objective of this study was to determine the agreement between gingivitis diagnoses generated using the 2018 classification (using BOP%) and clinicians' diagnoses using visual assessments such as changes in gingival color, volume, and stippling, indicating inflammation and BOP% (using both objective and subjective criteria). We determined if the BOP% criterion missed any patients who were diagnosed to have gingivitis through visual assessments and BOP%. To generate strong evidence on the agreement between the two diagnostic systems, we utilized a large electronic dental record (EDR) dataset from the patient examinations in the Indiana University School of Dentistry (IUSD) clinics. However, a manual review of this large data is not practical and can be error prone. Therefore, the second objective of this study was to create a computational program that automated gingivitis diagnoses from the EDR data and a natural language processing (NLP) program that retrieved diagnoses recorded in the clinical notes.

## Materials and methods

**Figure 1:**
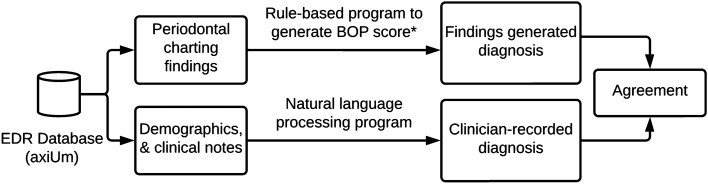
Stepwise methods to generate diagnoses from bleeding on probing (BOP) only, clinician-recorded diagnosis, and agreement calculation.

We conducted a retrospective study using the EDR data of patients who received at least one comprehensive oral evaluation (COE) in the IUSD predoctoral clinics. We developed a rule-based computational program to diagnose patients’ gingivitis statuses from periodontal charting findings (hereby referred to as BOP%-generated diagnoses), which are recorded as discrete data. An NLP was created to retrieve diagnoses recorded by clinicians in the clinical notes as free text (hereby referred to as clinician-recorded diagnoses). Next, we evaluated these programs’ performances and assessed the agreement between BOP%-generated diagnoses and clinician-recorded diagnoses. Finally, we determined the reasons for the agreement and disagreement between the BOP%-generated diagnoses and the clinician-recorded diagnoses (see Fig. 1). The study protocol was reviewed and approved by the Indiana University Institutional Review Board (#:1909819686, September 2019). Indiana University Institutional Review Board has waived the need for informed consent due to the retrospective nature of the study. Moreover, all methods were performed in accordance with the relevant guidelines and regulations presented by the Scientific Reports journal.

### Data set

EDR data (axiUm®, Exan software, Las Vegas, Nevada, USA) consisting of periodontal examination findings and charting of patients 18 years or older who received COE between January 1, 2009, and December 31, 2014, during the time of their first completed COE. We excluded those patients who came to the IUSD emergency clinics did not have a complete COE, who did not have any records (for example, paper or electronic records), and patients who did not have teeth (edentulous). We included 28 teeth (central incisors, lateral incisors, canines, first and second premolars, and first and second molars) and excluded third molar information from periodontal charting. This study time was used because we aimed to compare gingivitis diagnoses using subjective and objective criteria (prior to the release of 2018 criteria) versus only objective criteria. This paper only focuses on gingivitis, however, we also retrieved periodontal disease diagnoses^18^ using the same NLP approach to exclude patients who have gingivitis and/or periodontitis diagnoses recorded in the clinical notes. At IUSD, all patients receive a full-mouth hard and soft tissue examination during a COE. Patients’ soft tissue assessment included full-mouth visual assessment, periodontal charting, diagnoses, treatment planning, and prognosis assessment. Periodontal charting is recorded as structured data, while all other findings, including diagnoses, are recorded as free text in the clinical note referred to as the periodontal evaluation form (PEF).

Until 2018, student clinicians and their supervising faculty members diagnosed gingivitis status using visual assessments such as a change in gingival color, volume, stippling, and objective assessment of BOP%^5,19^. Using these criteria, they diagnosed patients as (1) localized or generalized gingivitis cases, and (2) severity status (mild, mild to moderate, moderate, moderate to severe, and severe). Since 2013, the Department of Periodontology at IUSD conducts regular calibration sessions of clinical faculty who supervise student clinicians^20,21^. During a COE, student clinicians record patients’ periodontal charting findings and diagnoses, which are reviewed, discussed and updated with a calibrated supervising faculty.

### Rule-based computational program to determine patients’ gingivitis from BOP%

We developed acomputational program (*Gingivitis_Diagnoser.py* see Python program as text file in supplementary material S1) that classified patients’ gingivitis status into healthy, localized, or generalized gingivitis cases based on 2018 BOP% and intact periodontium (see SM Table S1). *Gingivitis_Diagnoser.py* consisted of various Python inbuilt functions, variables, and rules such as *readlines ()*, *list ()*, *map ()*, *replace ()*, *len ()*, *search ()*, and *regular expressions ()*. First, the program created two temporary variables named *“Total_teeth”* and *“Total_Sites”* that determined the total number of teeth and number of sites present in a patient’s dentition respectively. For example, if the patient has 28 teeth present, then the total sites will be (28*6) 168 total sites which are stored in “Total_Sites” variable. Next, the program finds the total number of positive BOP sites in the entire dentition and stores this information in a temporary variable named *“Total_bleeding_sites”*. Finally, the program calculates the proportion of BOP sites (*“total_bleeding_sites”/” total_Sites”*). This program was applied to the entire dataset of 28, 908 patient records that contained a total of 17,104,308 observations consisting of clinical attachment loss, BOP, mobility, furcation involvement, tooth number and the six sites.

In summary, the program generates a total number of BOP sites by counting tooth sites with BOP findings recorded in the periodontal chart. The denominator (total number of sites “total_Sites”) is calculated by counting the total number of teeth and multiplying them by six because each tooth has six potential BOP sites. The program also estimates a tooth to be present if six probing depth sites per tooth were present in the charting (mesiobuccal, buccal, distobuccal, distolingual, lingual, and mesiolingual). Thus, if a patient has 48 BOP sites and 28 teeth with six probing depth sites, then this patient’s BOP score is 29% (48/28*6). Based on the BOP% criterion described in the 2018 classification 5, the patient is diagnosed to have a localized gingivitis case.

### NLP program to retrieve gingivitis and periodontal disease diagnoses from clinical notes

Clinicians typically document PD diagnoses in a textbox, in free text format, in the EDR. To retrieve diagnoses recorded as free text, we developed an NLP program (“Periodontal Disease Diagnoses Extractor.py” [see Python program as text file in supplementary materials S2] to retrieve patients’ PD diagnoses automatically in an analyzable structured format. First, to understand the diagnostic terms recorded by the clinicians, we reviewed 125 PEFs and annotated words and text phrases that indicated gingivitis or periodontitis diagnoses (see Table 1). Clinicians typically write PD type (gingivitis or periodontitis), severity (mild, mild to moderate, moderate, moderate to severe, and severe), location (maxilla, mandible, tooth number), and extent (localized or generalized). Typically, gingivitis and periodontitis diagnoses are recorded in the same field in the PEFs, therefore, our NLP program automatically extracts information for both gingivitis and periodontitis from the PEFs. The data set included 22,990 PEFs with a total word count of 145,173 containing diagnoses information. The average count for word per sentence was 7.20.

**Table 1:** Words or span of text in the electronic dental record notes that indicate gingivitis, and periodontal disease severity, and extent.

Gingivitis	Words or span of text
Disease type: gingivitis, periodontitis	gingivitis, inflammation of gingiva, periodontitis
Disease severity: mild, moderate, severe	mild, moderate, severe, mild to moderate, moderate to severe
Disease extent	localized, generalized

To retrieve a patients’ detailed PD information based on the disease type, severity, extension, location, and region, we used the approximate string-matching algorithm (ASM) from the Natural Language Toolkit (NLTK) library^22^. We also used the NLTK library to tokenize sentences and convert all words into lower cases. Tokenization is the process of splitting a text corpus into smaller segments that act as the first level of tokens. This process is also known as sentence segmentation because it attempts to segment the text into meaningful sentences or span of text. Then the text is further preprocessed by removing special characters and specific delimiters between sentences such as periods (.), newline characters (\n), and semi-colons (;). The ASM helps to find similar text or a directory even though spelling or grammatical errors are present in the text. The approximate string search is formulated to find the text or dictionary of size *“N”* of all the words that start or match with the given word while considering all the possible *“K” differential* errors. This algorithm works on the “*Levenshtein distance”* concept. The “*Levenshtein distance”* is a metric to measure the distance between two sequences of words. Typically, in this logic, a user is asked to set a percentage of the match per requirement. For example, if the word *“periodontitis”* is written with spelling errors such as “eriodontitis” or “poridontitis” the ASM logic can detect these variations present in the clinical text and identify them successfully. Similarly, the ASM algorithm was utilized to automatically extract a patient’s disease type, severity, location, and extent information.

### Performance of computational and NLP programs

Two faculty members (authors LW and DS (periodontist)) from the predoctoral comprehensive care clinic (hereby referred to as experts) calculated the BOP% using the 2018 gingivitis classification criterion^5^ (see SM Table S1). Each expert reviewer compared the manually calculated BOP% with the program’s output. After obtaining an excellent inter-rater agreement (Cohen’s Kappa statistic [25] = 1) for 50 patient cases, the two experts diagnosed gingivitis independently for 200 patient cases, which led to the development of a reference standard of 250 patient cases. The BOP%-generated diagnoses were also calculated using the program and compared with the reference standard. Precision, recall, and F-1 measure^23^ metrics were also calculated to assess the program’s performance (see SM Table S2).

The two experts also examined the diagnoses entered as free text in the PEF and the output generated by the NLP program. Using guidelines (see SM Tables S1–S5), they first reviewed 50 records and obtained excellent inter-rater agreement (Cohen’s Kappa = 1)^24^. Subsequently, each expert reviewed 150 records that resulted in 350 cases and constituted the reference standard. While evaluating NLP output with the free text in the PEF, the experts categorized each patient’s diagnoses into true positive, true negative, false positive, or false negative. A detailed description of the manual review process with examples are provided in the supplementary materials Sect. 2 and SM Tables S3–S7. Next, we compared the NLP program results and the experts’ assessment and calculated precision, recall, and F-1 score^23^ for the program.

### Final data set

**Figure 2:**
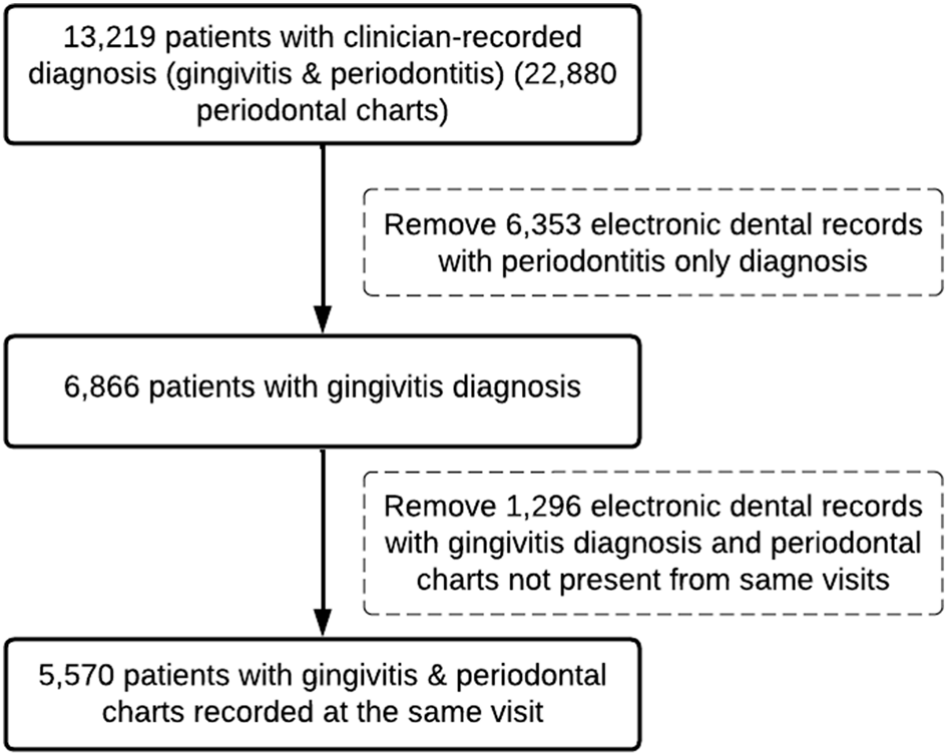
Generation of the final dataset to determine the agreement between gingivitis diagnoses generated from bleeding on probing (BOP) only and clinician-recorded diagnoses documented during the same comprehensive oral evaluation (COE).

After applying the rule-based and NLP programs, we obtained patients’ gingivitis diagnoses from the charting and patients’ clinician-recorded gingivitis and periodontitis diagnoses from the clinical notes. Next, we excluded the EDR of patients who had periodontitis or both gingivitis and periodontitis recorded in the clinical notes. The final data set included EDR data that had clinician-recorded diagnoses of gingivitis, including healthy gingiva and a clinical attachment loss of less than 4 mm indicating intact periodontium (see Fig. 2); in addition, it included only EDR data of patients who had periodontal charting clinician-recorded diagnoses from the same visit date.

### Agreement between BOP%-generated diagnoses and clinician-recorded diagnoses

According to Weiskopf et al.^25^, data is considered concordant when there is an agreement of compatibility between the same information present in two data fields. We calculated the percent agreement between BOP%-generated gingivitis diagnoses and clinician-recorded diagnoses. The agreement was determined for a patient’s disease status (healthy vs. gingivitis) and disease extent (localized vs. generalized).

The two experts reviewed 145 randomly selected patient records to determine the reasons for agreement and disagreement between BOP%-generated and clinician-recorded diagnoses. Out of 145 records, 50 were reviewed by both experts to ensure high inter-rater reliability (Cohen’s Kappa score of 0.9 (excellent rating)). Through discussion and consensus, the experts determined the reasons for agreement/disagreement between the two diagnoses. They also reviewed notes recorded in the PEF to ensure the clinician-recorded diagnoses was accurate and was determined to have inter-rater reliability of 0.86 (Cohen’s Kappa statistic for inter-rater agreement). This high agreement provided excellent accuracy of the clinician-recorded diagnoses. Therefore, we considered this manually evaluated dataset as a gold-standard dataset because the patient is extensively examined by the student, periodontal resident, faculty member, and the two experts (total five clinicians). The clinical note recorded in the PEF provided comprehensive information about patients’ gingival health including the bleeding on probing values recorded during periodontal charting.

### Data analysis

Descriptive statistics were calculated with a 95% confidential interval for demographics variables such as age, gender, race, and insurance status by clinician-recorded diagnoses and BOP%-generated diagnoses. Age was stratified by 18–29, 30–44, 45–64, and 65 years and greater as reported in the national prevalence study by Eke et al.^26^ Patients’ gender, race, and insurance status were classified as displayed in Table 2. Percent agreement was calculated to determine the agreement between clinician-recorded diagnoses and the BOP%-generated diagnoses.

**Table 2:** Differences in the number of patients by demographics among 5570 patients who have a gingivitis diagnoses recorded in the electronic dental record (EDR) (clinician-recorded diagnoses) versus the number of patients whose diagnoses is calculated using bleeding on probing score (BOP%) based on the 2018 diagnostic classification for gingivitis (BOP%-generated diagnoses) (N = 5570).

Patient characteristics	Clinician-recorded diagnoses		BOP score-generated diagnoses	
Age (years)				
18–29	1958	(35.2)	703	(12.6)
30–44	1589	(28.5)	619	(11.1)
45–64	1519	(27.23)	451	(8.1)
65 and greater	504	(9)	133	(2.4)
Gender				
Female	3138	(56.3)	1043	(18.7)
Male	2403	(43.1)	853	(15.3)
Transgender	1	(0)	1	(0)
Other	28	(0.5)	9	(0.2)
Race/ethnicity				
White	2906	(52.2)	952	(17.1)
Black or African American	691	(12.4)	252	(4.5)
Hispanic	374	(6.7)	168	(3)
Asian	147	(2.6)	53	(1)
Other	26	(0.5)	12	(0.2)
Multiracial	4	(0.1)	1	(0)
American Indian	1	(0)	1	(0)
Unknown	1420	(25.5)	467	(8.4)
Insurance				
Self-pay	2887	(51.8)	1011	(18.2)
Private	2315	(41.6)	743	(13.3)
Government	367	(6.6)	152	(2.7)
Patients with healthy gingiva	0	(0)	3663	(65.8)
Total	5570	(100)	5570	(100)

### Ethical approval

This project was approved by the Indiana University Institutional Review Board (#1909819686) as Exempt research.

### Contribution to the field statement

This study demonstrated that a significant percent of people with BOP%<10% on an intact periodontium could also have visual signs of gingival inflammation such as redness and swelling, which is considered periodontally healthy according to the 2018 diagnostic classification. Given that visual signs have been a significant part of diagnosing gingivitis until 2018, periodic training and calibration exercises are necessary for clinicians and researchers to transition them to using the new classification consistently in their practice.

## Results

### Patient characteristics and demographics

The EDR data included 28,908 distinct patients who received at least one COE between January 1, 2009, and December 31, 2014. The mean age of patients was 46 years (standard deviation = 16.74), and fifty-four percent were females (see SM Table S8). Seventy-nine percent (n = 22,880) of patients had at least one full-mouth periodontal charting, and 46% (n = 13,219) of patients had both clinician-recorded diagnoses and full-mouth periodontal charting available in the EDR (see Fig. 2).

### Clinician-recorded (gingivitis and periodontitis) diagnoses from clinical notes and BOP%-generated diagnoses generated from BOP%

The clinician-recorded diagnoses were available for a total of 13,219 patients (46%) and BOP%-generated diagnoses were available for 80% of patients (see Fig. 2). Among these patients, 3193 patients (24%) were diagnosed (clinician-recorded diagnoses) with mild gingivitis, 1607 (12%) with moderate gingivitis, and 143 (1%) with severe gingivitis (see Table 3). Eighteen percent of patients (2430) were diagnosed with mild periodontitis, 1899 (14%) with moderate periodontitis, and 554 (4%) with severe periodontitis cases (see Table 3). Clinicians also recorded patients’ PD diagnoses with two additional severity categories such as “mild to moderate”, and “moderate to severe”. There were 247 (2%) patients with mild to moderate gingivitis, 62 (0.5) with moderate to severe gingivitis, 569 (4%) with mild to moderate periodontitis and 350 (3%) with moderate to severe periodontitis. In addition, 1613 cases were classified as gingivitis, and 258 patients as periodontitis because of the lack of availability of these patients’ disease severity information. After excluding patients with periodontitis, the final study data set consisted of 5570 patients with clinician-recorded gingivitis on intact periodontium (see Fig. 2). The mean age of patients with gingivitis diagnoses was 40 years (standard deviation = 15.79), with 56% females, 52% White, and 52% self-pay patients (see Table 2). Detailed classification of gingivitis diagnoses using the periodontal charting data is displayed in Table 4.

**Table 3:** Clinician-recorded gingivitis and periodontitis diagnoses documented by clinicians in the periodontal evaluation form as free text.

Gingivitis/periodontitis diagnoses	N	(%)
Mild gingivitis*	3,193	(24)
Mild to moderate gingivitis*	247	(2)
Moderate gingivitis*	1,607	(12)
Moderate to severe gingivitis*	62	(0.5)
Gingivitis*	1,613	(12)
Severe gingivitis*	143	(1)
Mild periodontitis	2,430	(18)
Mild to moderate periodontitis	569	(4)
Moderate periodontitis	1,899	(14)
Moderate to severe periodontitis	350	(3)
Periodontitis	258	(2)
Severe periodontitis	554	(4)
Missing/no disease mentioned/algorithm error	294	(2)
Total (available data)	13,219	(100)
Missing data	15,689	(54)
Total	28,908	(100)

*Patients diagnosed with gingivitis is 6,865 (greater than 5570) because it includes patients with gingivitis and periodontitis diagnoses recorded as free text. However, for the final data set (Table 2), only clinician-recorded gingivitis diagnoses that also had periodontal charting during the same visit is included. (N = 28,908).

**Table 4:** Patients' gingivitis diagnoses determined from the bleeding on probing score (N = 5570).

Gingivitis	N	(%)
Healthy	3663	(66)
Localized	1476	(26)
Generalized	430	(8)
Unknown	1	(0)
Total	5570	(100)

### Agreement between BOP%-generated and clinician-recorded gingivitis diagnoses in the final dataset

The agreement between the BOP%-generated and clinician-recorded gingivitis diagnoses was 34% (1894 patients) for the patients’ disease status. Sixty-six percent (3663 patients) of clinician-recorded gingivitis cases out of 5570 patients were diagnosed as “healthy” when considering the 2018 classification (BOP% < 10%).

The agreement changed to 9% when disease status and extent were considered together in 5099 of the 5570 patients. The two experts found that the clinician-recorded gingivitis diagnoses for every patient was based on the clinician’s visual assessment of the soft tissue, such as a change in gingival color, volume, and stippling indicating gingival inflammation. However, most patients’ BOP% did not meet the 2018 diagnostic criterion to be diagnosed as a gingivitis case with intact periodontium.

### Performance of computational and NLP programs

The rule-based program that automated the BOP% calculation demonstrated excellent performance of 99% precision, 100% recall, and 99.5% F-measure. Similarly, the NLP program demonstrated an average of 98% precision, recall, and F-1 measure to retrieve patients’ disease status, severity, extent, and location from the clinic notes (see Table 5).

**Table 5:** Performance of the natural language processing program (N = 350).

Concepts	Precision (%)	Recall (%)	F-measure (%)
Disease status (gingivitis)	99	99	99
Disease extent (localized/generalized)	99	99	99
Disease region	97	97	97
Disease severity	97	96	96
Overall	98	98	98

## Discussion

The number of patients diagnosed with gingivitis as defined by the 2018 classification using BOP%^5^ was sixty-six percent less compared to patients diagnosed through visual assessment and BOP%^19^. The results confirm that visual signs of inflammation such as changes in gingival color and volume could be present in a healthy gingiva patient case with a BOP% < 10%. Although the 2018 classification for gingivitis is objective and simple to use, the results indicate that considerable variations in diagnoses and treatment planning among clinicians can occur as they use this new system. A recent study^11^ that assessed practitioners’ understanding of the 2018 PD classification system reported that the gingivitis case proved to be the most challenging, with 65–95% of the periodontal specialists and general practitioners incorrectly diagnosing a healthy gingiva case as biofilm induced gingivitis. Our study results quantify the extent to which soft tissue inflammatory changes could be present among healthy gingiva cases. This finding could facilitate dental clinicians and researchers to diagnose a patient case with BOP% < 10% as healthy even in the presence of visual signs of gingival inflammation. Moreover, training and calibration sessions are necessary to enable consistent use of the new diagnostic classification. Lastly, the computational approaches that generated diagnoses from findings and retrieved diagnostic information from clinical notes enabled studying a larger population of patient data. These programs could be used in the future to study the prognosis and change in disease status among patients diagnosed with gingivitis and healthy gingiva using the 2018 diagnostic classification. Below, we discuss the key points from the study in detail.

### Differences in diagnosing gingivitis using BOP% versus visual assessment and BOP%

Patients classified as having healthy gingival tissues could actually have up to 9% (“< 10%”) BOP% with inherent gingival inflammation exhibiting signs such as swelling, redness, and tenderness on palpation. We understand that one of the rationales for the new diagnostic criteria was to differentiate between having a few “gingivitis sites” versus being described as a “gingivitis case”. However, the inconsistencies in clinicians’ and researchers’ diagnoses using the new classification system could lead to under- or over-estimation of the disease^6,11^. Gingivitis is a precursor of periodontitis and can be reversed if diagnosed early. Thus, it is important to diagnose and manage gingivitis early to prevent progression to periodontitis. Therefore, periodic training and calibration of clinicians and researchers are critical to facilitate the correct use of the new diagnostic system even if these criteria are easy to use and objective measures. Inaccuracies in the diagnoses could limit the advantages of using the new diagnostic system.

### Using EHR data to validate case definitions and determining EDR data quality

As described before, studies in medicine^16,17^ have validated various disease classifications using the EHR data of a large population. Similarly, our study illustrates the strength of using EDR data to study the effect of new case definitions that may be difficult to pursue through prospective studies^27^. Despite the promising potential of using EDR data for research, it is important to evaluate the quality of the EDR data before its intended use since the EDR data is not collected for research, but for patient care^28,29^. In this study, we determined the accuracy of the clinician-recorded diagnoses by comparing with expert reviewers’ diagnoses. The results demonstrated excellent agreement indicating high accuracy and reliability of the gingivitis diagnoses information documented in EDR. This high data quality can be attributed to the regular training and calibration sessions on diagnoses and treatment planning performed among faculty and residents in the IUSD Department of Periodontology^21^. The high reliability of EDR data indicates the importance of incorporating training and calibration sessions for dental clinicians as part of their discipline and continuing education to enhance consistency in clinical assessment and documentation across different clinical settings.

### Role of computational methods to process large-size data and retrieve findings from clinical notes

This study required processing approximately 12 million observations at the rate of at least 600 periodontal charting observations per patient using computational approaches. In addition, we used text mining approaches to retrieve diagnoses written in the clinical notes. Thus, this study would not have been possible without using computational approaches to process such large-size data accurately and efficiently. Manual processing of such big data would have required considerable time and effort with the potential for errors. Previous studies^28,30,31^ have reported retrieving periodontal data and radiographs to assess change in disease status, data completeness and radiographic bone loss. Nevertheless, the computational and NLP approaches developed in this study are valuable resources for researchers and clinicians to determine PD diagnoses from periodontal data as well as from EDR clinical notes. It is possible to apply these automated approaches developed in this study to different datasets and private practice data to determine the national prevalence of gingivitis like the NHANES studies. This approach could be an alternative to the NHANES studies because NHANES reported not performing prevalence studies due to the high cost involved in conducting clinical examination in a large population^26^.

### Limitations and future work

Like any study, limitations exist in this study. First, we included patients who only had gingivitis and not periodontitis, as the current classification discriminates gingivitis between patients with intact periodontium and reduced periodontium. Our aim was to determine the percentage of patients with healthy gingiva diagnosed using the 2018 classification 5 who also have the visual signs of inflammation previously used to diagnose gingivitis^5,19^. Next, the study dataset included only patients who received COE between January 1, 2009 and December 31, 2014. We chose this time because our long-term goal is to compare our patient populations’ periodontitis prevalence with the national prevalence used at the same time. Another limitation is that the patient data were collected from student clinics with a potential for variations in certain findings even though calibrated faculty review the findings. Next, we did not differentiate plaque-induced gingivitis from the non-plaque induced gingivitis. Further, we excluded periodontal charting information collected on patients’ third molars that could be treated for gingivitis. Lastly, the inclusion of EDR data from one institution limited the generalizability of the results. In the future, we will differentiate plaque induced gingivitis from non-plaque induced gingivitis cases. We will also determine the influence of other factors such as smoking on the BOP outcome in gingivitis patients.

### Conclusions

This study demonstrated a significant number of patients, diagnosed with healthy gingiva according to the 2018 diagnostic classification, could have the visual signs of inflammation such as the change in gingival color (redness) and volume (swelling). The results highlight the potential for variations among clinicians when diagnosing gingivitis using this new classification, thus, leading to under- or over-estimation of gingivitis cases. Periodic training and calibration of clinicians, dental students and researchers can lead to consistent use of the new diagnostic system. The study also demonstrated the significance of utilizing EDR data for PD research. The informatics approaches developed in this study provide a basis to apply patient care data in other academic institutions and community practices to generate diagnoses from periodontal charting data and retrieve diagnoses recorded as free text in the clinical notes. Finally, we also conclude that the calibration practices at our institution significantly improved the consistency of PD diagnoses and treatment planning. The results of this study indicate that training and calibration sessions could lead to better documentation of patient information in the EDR.

### Supplementary Information

Supplementary Information 1.

Supplementary Information 2.

Supplementary Information 3.

## Data Availability

The datasets generated and/or analyzed during the current study are not publicly available because the dataset contains identifiable information. Therefore, permission is not granted to share publicly but are available from the corresponding author on reasonable request.
