# Supplementary material for: Comparing gingivitis diagnoses by bleeding on probing (BOP) exclusively versus BOP combined with visual signs using large electronic dental records
Source: Sci Rep. 2023 Oct 10;13:17065. doi: 10.1038/s41598-023-44307-z (PMC10564949; doi:10.1038/s41598-023-44307-z)
Supplement: Supplementary file 1 — Supplementary Information 1. [file 41598_2023_44307_MOESM1_ESM.pdf]

```
import os,re
def calculate_total_sites(file_content):

    total_sites = 0
    sites_used = [] # list that stores all of the sites that have been read only once
    for i in range(len(file_content)): # Runs the following code for every line in the data
        if 'SiteName:' in file_content[i]: # Checks if the keyword 'SiteName:' exists in the line. If yes: performs the code 
below
            current_site = file_content[i][10:] # Creates variable 'current_site' that stores the sitename eg: 10
            if not current_site in sites_used: # Checks whether the current_site variable exists in the 'sites_used' list
                sites_used += [current_site]
                total_sites += 1 # Adds 1 to the previous value of the variable 'total_sites'
    return total_sites * 6

def calculate_bop(file_content):
    """
    This function reads the file and checks for the number of sites that were bleeding and then returns it.
    """
    bop_sites = 0
    for i in range(len(file_content)):
        if 'PerCond: BLEED' in file_content[i]:
            values = [file_content[i+3][8:],file_content[i+4][8:],file_content[i+5][8:]]
            for item in values:
                if item == '1' or item == 'B' or item == 'b':
                    bop_sites += 1

    return bop_sites

def calculate_attach_sites(file_content):
    """
    This function reads the file and checks for the number of attach sites and returns it
    """
    attach_sites = 0
    for i in range(len(file_content)):
        if 'PerCond: ATTACH' in file_content[i]:
            attach_sites += 1
    return attach_sites



def diagnose(total_sites, bop_sites,file):
    """
    This function calculates the percentage of the number of sites that bled and uses the given criteria to move the file 
into
    its determined diagnosis.
    """
    if total_sites == 0: # Checks if the text file didn't contain any sites meaning it was an invalid file and moves it to the 
Unknown folder
        os.rename(file,'Unknown/'+file)
        return "Unknown"

    elif round((bop_sites / total_sites) * 100) < 10: # Checks if the bop score is less than 10% if so: moves it to 'No 
Gingivitis' folder.
        os.rename(file,'No_Gingivitis/'+file)
```

```
        return "No Gingivitis"
    elif round((bop_sites / total_sites) * 100) >= 10 and round((bop_sites / total_sites) * 100) <= 30: # Checks if the bop
score is less than or equal to 30% and also greater than or equal to %10 if so: moves it to 'Localized Gingivitis' folder.
        os.rename(file,'Localized_Gingivitis/'+file)
        return "Localized Gingivitis"
    elif round((bop_sites / total_sites) * 100) > 30: # Checks if the bop score is more than 30% if so: moves it to
'Generalized Gingivitis' folder.
        os.rename(file,'Generalized_Gingivitis/'+file)
        return "Generalized Gingivitis"

def main():
    files = []
    try: # Tells the program not to crash if it is unable to create the folders
        os.mkdir('No_Gingivitis')
        os.mkdir('Localized_Gingivitis')
        os.mkdir('Generalized_Gingivitis')
        os.mkdir('Unknown')
    except FileExistsError:
        pass

    for f in os.listdir():
        if re.search('.txt',f):
            files += [f]

    log_file_content = ""
    for file in files: # Performs the following code for every text file contained in the directory
        data = open(file,'r').readlines() # Reads the data from the text file and stores it in a variable called 'data'
        data = list(map(str.strip,data)) # Removes the newline characters from the 'data' variable
        patient_id = file.split('_')[0] # Gets the first item contained in the line which is patient id and stores it in a variable
called 'patient_id'
        patient_date = file.split('_')[1].replace('.txt','')
        total_sites = calculate_total_sites(data)
        total_teeth = round(total_sites / 6)
        bop_sites = calculate_bop(data)
        attach_sites = calculate_attach_sites(data)
        if attach_sites == 0: # If the text file is missing attach information, move the file to the unknown folder.
            log_file_content += ("Patient ID: " + str(patient_id) + "\n" + "Date: " + str(patient_date) + "\n" + "Total Teeth:
ATTACH MISSING" + "\n" + "Total Sites: ATTACH MISSING" + "\n" + "BOP Sites: ATTACH MISSING" + "\n" +
"Affected: ATTACH MISSING"+ "\n" + "Diagnosis: UNKNOWN" + "\n\n\n")
            os.rename(file,'Unknown/'+file)
            continue
        diagnosis = diagnose(total_sites,bop_sites,file)

        log_file_content += ("Patient ID: " + str(patient_id) + "\n" + "Date: " + str(patient_date) + "\n" + "Total Teeth: " +
str(total_teeth) +"\n" + "Total Sites: " + str(total_sites) + "\n" + "BOP Sites: " + str(bop_sites) + "\n" + "Affected: " +
str(round((bop_sites / total_sites) * 100)) +"%"+ "\n" + "Diagnosis: " + diagnosis + "\n\n\n")
    log_file = open("log.txt",'w') # Creates a log file containing each patientsâ€™ information and the diagnosis
    log_file.write(log_file_content)
    log_file.close()

if __name__=="__main__":
    main()
```
