# Supplementary material for: Comparing gingivitis diagnoses by bleeding on probing (BOP) exclusively versus BOP combined with visual signs using large electronic dental records
Source: Sci Rep. 2023 Oct 10;13:17065. doi: 10.1038/s41598-023-44307-z (PMC10564949; doi:10.1038/s41598-023-44307-z)
Supplement: Supplementary file 2 — Supplementary Information 2. [file 41598_2023_44307_MOESM2_ESM.pdf]

```
"""

import nltk
import string
import re
import pandas as pd

global_loc=[]
global_reg=[]
global_disease=[]
global_time=[]
global_severity=[]
global_id=[]
global_diagnosis=[]
global_date=[]
global_birth=[]
global_race=[]
global_sex=[]
global_insurance=[]

def get_time_period(words):
  tp=''
  labels = ['chronic', 'acute']
  dic = {}
  for l in labels:
     dic[l] = 0
  for w in words:
     for l in labels:
        ed = nltk.edit_distance(l, w)
        if ed < 3:
          # print('ed ={}'.format(l))
           dic[l] += 1
  if dic['chronic'] >= 1:
     tp= 'chronic'
  elif dic['acute'] >= 1:
     tp = 'acute'
  else:
     tp='No time period'
  return tp

def get_disease(words):
  disease = ''
  labels = ['gingivitis','periodontitis']
  dic = {}
  for l in labels:
     dic[l] = 0
  for w in words:
     for l in labels:
        ed = nltk.edit_distance(l, w)
        if ed < 5:
           #print('ed ={}'.format(l))
           dic[l] += 1
  if dic['gingivitis']>=1:
```

```
        disease='gingivitis'
    elif dic['periodontitis']>=1:
        disease='periodontitis'
    else:
        disease='No disease specified'

    return disease

def get_severity(words):
    severity=''
    labels = ['mild', 'moderate', 'severe']
    dic = {}
    for l in labels:
        dic[l]=0
    for w in words:
        for l in labels:
            ed=nltk.edit_distance(l,w)
            if ed<3:
                # print(w)
                # print('ed ={}'.format(l))
                dic[l]+=1
    if dic['mild']>=1 and dic['moderate']>=1:
        severity='mild to moderate'
    elif dic['moderate']>=1 and dic['severe']>=1:
        severity='moderate to severe'
    elif dic['mild']==1:
        severity='mild'
    elif dic['moderate']==1:
        severity='moderate'
    elif dic['severe']==1:
        severity="severe"
    else:
        severity = 'no abnormality'

    return severity

def get_reg(words):
    region_labels = ['maxillary', 'mandibular']
    for w in words:
        for l in region_labels:
            ed =nltk.edit_distance(l, w)
            if ed < 4:
                return l
    teeth=[]
    for w in words:
        '''if '-' in w or '&' in w:
            if '-' in w:
                s_range=w.split('-')
            else:
                s_range=w.split('&')
            if is_number(s_range[0]) and is_number(s_range[1]):
                for i in range(int(s_range[0]),int(s_range[1])+1):
                    teeth.append(i)'''
        if hasNumbers(w):
```

```
        teeth.append(w)
    if len(teeth)>0:
        return teeth

    return 'No region specified'

def get_loc_pos(words):
    loc_labels = ['generalized', 'localized']
    location_list = []
    for i, w in enumerate(words):
        for l in loc_labels:
            ed =nltk.edit_distance(l, w)
            if ed < 4:
                location_list.append(i)
    return location_list

def get_loc(words):
    loc_labels = ['generalized','localized']
    location_tuples=[]
    for i,w in enumerate(words):
        for l in loc_labels:
            ed = nltk.edit_distance(l, w)
            if ed < 4:
                return l
    return 'No location specified'

def isNan(x):
    return x!=x

def hasNumbers(inputString):
    return any(char.isdigit() for char in inputString)

def is_number(s):
    try:
        float(s)
        return True
    except ValueError:
        return False

def read_xcel():
    df = pd.read_excel('try.xlsx')
    print(df.columns)
    #print(df['Id'])
    for i,s in enumerate(df['diagnosis']):
        if isNan(s)==False:
            #print(df['Id'][df.index[i]])
            clean(s,df['Id'][df.index[i]],df['Date'][df.index[i]],df['birth'][df.index[i]],df['sex'][df.index[i]],df['race']
[df.index[i]],df['Insurance'][df.index[i]])


def clean(diag,pid,date,birth,sex,race,insurance):
    words = re.split(r'[^a-zA-Z0-9_&]', diag)
    pat=re.compile('\d+')
    numbers=pat.findall(diag)
```

```
    # table = str.maketrans('', '', string.punctuation)
    # words = [w.translate(table) for w in words]
    words=[word.lower() for word in words]
    #stop_words = set(stopwords.words('english'))
    stop_words={'needn', 'mightn', 'a', 'not', 'then', 'ours', 'wouldn', 'those', 'our', "doesn't", 'having', 'again', 'most',
            'mustn', 'his', 'd', 'below', 'when', 'only', "isn't", 've', "mightn't", 'during', "you'll", 'is', 'can', 'couldn', "wasn't",
            'were', 'at', 'both', 'by', 'other', 'about', "you're", 'some', 'ain', 'your', 'yours', 'hasn', 'until', 'above',
            'you', 'very', 'few', 'herself', 'they', 'on', "mustn't", 'why', 'didn', 'no', "needn't", 'themselves', 'should',
            'shouldn', 'aren', 'don', 'shan', 'himself', 'or', 'it', 'as', 'so', 'did', 'she', 'and', 'hers', 'ma', 'll', "won't",
            "should've", 'where', 'the', 'over', "don't", 'who', 'off', 'we', 'all', 'if', 's', 'its', 'any', 'than', 'me', "weren't",
            'am', 'do', 'there', 'here', 'which', "you'd", 'because', 'was', 'weren', 'being', 'be', 'further', 'each', 'whom', 'her',
            'out', "shan't", 'an', 'haven', 'in', 'been', 'under', 'same', 'o', 'theirs', "that'll", "you've", 'but', 'y', 'won', 'i',
            'to', 'nor', 'them', 'against', 'for', "couldn't", 'yourselves', 'these', "hasn't", 'now', 't', 'own', 'between', 'up', "it's",
            're', 'has', 'doesn', 'how', 'such', "wouldn't", "didn't", 'through', "haven't", 'isn', "shouldn't", 'he', 'my', 'him',
"hadn't",
            'yourself', 'while', 'will', 'ourselves', 'their', 'itself', 'what', 'does', 'are', 'have', 'into', 'wasn', 'of', 'hadn', "she's",
            'doing', 'after', 'once', 'm', 'this', 'had', 'more', 'that', 'just', 'down', "aren't", 'with', 'from', 'before', 'myself', 'too'}
    words = [w for w in words if not w in stop_words and len(w)>0]
    loc_list=get_loc_pos(words)
    last_end=0
    for start,end in zip(loc_list,loc_list[1:]):
        location=words[start]
        my_print(words[start:end+1],diag,pid,date,birth,sex,race,insurance)
        last_end=end
    my_print(words[last_end:],diag,pid,date,birth,sex,race,insurance)

def my_print(words,diagnosis,pid,date,birth,sex,race,insurance):
    disease = get_disease(words)
    severity = get_severity(words)
    time_period = get_time_period(words)
    reg=get_reg(words)

    location=(get_loc(words))
    print('location={}'.format(location))
    print('region={}'.format(reg))
    print('disease={}'.format(disease))
    print('severity={}'.format(severity))
    print('time period ={}'.format(time_period))


    global global_loc
    global global_reg
    global global_disease
    global global_time
    global global_severity
    global global_id
    global global_diagnosis
    global global_birth
    global global_sex
    global global_race
    global global_insurance
    global global_date

    global_loc.append(location)
```

```
    global_reg.append(reg)
    global_disease.append(disease)
    global_time.append(time_period)
    global_severity.append(severity)
    global_id.append(pid)
    global_diagnosis.append(diagnosis)
    global_birth.append(birth)
    global_sex.append(sex)
    global_race.append(race)
    global_insurance.append(insurance)
    global_date.append(date)


def main():
    writer = pd.ExcelWriter('cleaned_diagnosis_small.xlsx')
    read_xcel()

n_df=pd.DataFrame({'pid':global_id,'diagnosis':global_diagnosis,'disease':global_disease,'time':global_time,'severity':gl
obal_severity,'location':global_loc,'region':global_reg,'birth':global_birth,'sex':global_sex,'race':global_race,'insurance':g
lobal_insurance,'date':global_date})
    #print(n_df)
    n_df.to_excel(writer)
    writer.save()

if __name__ == '__main__':
    main()



import nltk
import string
import re
import pandas as pd
from collections import defaultdict

df = pd.read_excel('structured_diagnosis_final.xlsx')
d=defaultdict(list)
time=defaultdict(list)
for i,v in enumerate(df['pid']):
     s=str(df.iloc[i]['location'])+' '+str(df.iloc[i]['severity'])+' '+str(df.iloc[i]['disease'])+' '+str(df.iloc[i]['time'])
     d[v].append(s)
     date=str(df.iloc[i]['date'])
     d[v].append(date)

writer = pd.ExcelWriter('output_progression_structured.xlsx')

n_df=pd.DataFrame(list(d.items()),columns=['pid','td'])
n_df2=pd.DataFrame(n_df.td.values.tolist())
n_df2['pid']=list(d.keys())



df=df.drop(labels=['diagnosis','disease','location','region','severity','time','date'],axis=1)
#
df=df.drop_duplicates(subset='pid')
```

```
df=df.set_index('pid')
n_df2=n_df2.join(df,on='pid')


n_df2.to_excel(writer)
writer.save()
```

Putting clinician-recorded diagnoses in buckets.

```
# -*- coding: utf-8 -*-
"""
Created on Tue Jul 17 08:27:11 2018

@author: kumarkr
"""

import nltk
import string
import re
import pandas as pd
from collections import defaultdict

df = pd.read_excel('cleaned_diagnosis_small.xlsx')
d=defaultdict(list)
time=defaultdict(list)
for i,v in enumerate(df['pid']):
    s=str(df.iloc[i]['location'])+' '+str(df.iloc[i]['severity'])+' '+str(df.iloc[i]['disease'])+' '+str(df.iloc[i]['time'])
    d[v].append(s)
    date=str(df.iloc[i]['date'])
    d[v].append(date)

writer = pd.ExcelWriter('bin_diagnosis_small.xlsx')

n_df=pd.DataFrame(list(d.items()),columns=['pid','td'])
n_df2=pd.DataFrame(n_df.td.values.tolist())
n_df2['pid']=list(d.keys())



df=df.drop(labels=['diagnosis','disease','location','region','severity','time','date'],axis=1)
#
df=df.drop_duplicates(subset='pid')
df=df.set_index('pid')
n_df2=n_df2.join(df,on='pid')


n_df2.to_excel(writer)
writer.save()
```
