# Supplementary material for: Comparing gingivitis diagnoses by bleeding on probing (BOP) exclusively versus BOP combined with visual signs using large electronic dental records
Source: Sci Rep. 2023 Oct 10;13:17065. doi: 10.1038/s41598-023-44307-z (PMC10564949; doi:10.1038/s41598-023-44307-z)
Supplement: Supplementary file 3 — Supplementary Information 3. [file 41598_2023_44307_MOESM3_ESM.docx]

Supplementary Material (SM)

**Comparing Gingivitis diagnoses by bleeding on probing (BOP) exclusively versus BOP combined with visual signs using large electronic dental records**

Content:

1. Section 1:

Objective: Description of the study data files

Manual review and determining performance of the program

SM Table 1: Diagnostic criteria for gingivitis

SM Table 2: Formulas used to determine the performance of computational program: gingivitis_diagnoser.py

1. Section 2:

Objective: Manual review process and evaluation of the NLP performance

SM Table 3: An example of a true positive case output of the NLP program for a clinician- recorded diagnosis of “mild chronic generalized periodontitis”

SM Table 4: Example of a false positive case output by the NLP program for a clinician-recorded diagnosis of “mild generalized gingivitis”

SM Table 5: Examples of false positive case outputs by the NLP program

SM Table 6: Example of a false negative output for a clinician-recorded diagnosis of “mild generalized gingivitis”

SM Table 7: Possible examples to correctly identify false negative cases

SM Table 8: Demographics of patients who received at least one comprehensive oral evaluation at Indiana University School of Dentistry between January 1, 2009, and December 31, 2014

# Section 1: Manual review guidelines to evaluate performance of the gingivitis diagnoser.py (listed as text file) program

**Objective:** The objective of this manual review process was to evaluate the performance of the gingivitis diagnoser.py program that automatically generated gingivitis diagnosis from periodontal charting findings.

**Description of the study data files:** Each data file manually reviewed by the experts had the following information:

- patient ID
- appointment date
- periodontal chart that contains probing depths, clinical attachment loss, and bleeding on probing
- diagnosis generated automatically by gingivitis diagnoser.py program based on periodontal chart findings.

**Manual review and determining performance of the program**

First, experts reviewed 50 patients’ charting information and diagnosed their gingivitis status based on the criteria described in Table 1. The inter-rater agreement between the experts was 0.9 (Cohen’s Kappa statistic) which indicated excellent agreement. Next, each expert reviewed 150 patient records independently, which resulted in a total of 350 patient records. The gingivitis diagnosis generated automatically from the 350 patient records by the program gingivitis_diagnoser.py was compared for agreement with the diagnosis recorded by the experts. Based on the computer algorithm’s ability to diagnose gingivitis cases correctly, true positive, false positive, and false negative were calculated. Using these measures, precision, recall, and f-measure were calculated (see Table 2) to determine the performance of gingivitis_diagnoser.py.

SM Table 1: Diagnostic criteria for gingivitis

| **Disease status** | **Rules** |
| --- | --- |
| No gingivitis | Absence of “B” or “1” corresponding to “BLEED” value in the charting text file OR BOP score is <10%. |
| Localized gingivitis | BOP score is >= 10% AND <=30%. |
| Generalized gingivitis | BOP score is > 30%. |

**SM Table 2: Formulas used to determine the performance of computational program: gingivitis_diagnoser.py**

| **Evaluation measures** | **Formulas** |
| --- | --- |
| Precision | true positive / (true positives + false positives) |
| Recall | true positives / (true positive + false negatives) |
| F-measure | 2 * (precision * recall) / (precision + recall) |

For the manual review, we created a gingivitis validation calculator in Microsoft Excel^®^. Experts first manually counted the total number of bleeding sites in the charting file and then manually entered this information in the BOP sites. If there are no bleeding sites, then they considered bleeding sites = 0. Next, they calculated total number of teeth by manually reviewing the number of teeth that had CAL information recorded. Next, they determined the BOP score by dividing the total number of bleeding sites by total number of sites (total teeth * 6 probing sites).

# Section 2: Manual review guidelines to assess the performance of the Natural Language Processing Program

**Objective:** Determine the performance of the natural language processing (NLP) program developed to retrieve clinician-recorded diagnosis from clinical notes (periodontal evaluation form) in a structured format.

**Manual review process and evaluation of the NLP performance**

To determine the program’s performance, first, expert reviewers (co-authors DS and LW) compared the output generated by the NLP program with the diagnosis recorded (hereby referred as clinician-recorded diagnosis) in the periodontal evaluation form in the patients’ electronic dental record. As described in the paper, clinicians document the following information regarding periodontal disease (PD) diagnosis:

- PD type: Gingivitis or periodontitis
- Disease severity: Mild, mild to moderate, moderate, moderate to severe, severe
- Disease extent: Localized or generalized
- Disease location: Maxilla, mandible, tooth number.

For example, diagnosis is recorded as “chronic generalized mild to moderate periodontitis.” The NLP program classified a patient’s PD diagnosis based on PD type, severity, onset, extent and location. The reviewers classified the program’s output into true positive, false positive, and false negative categories based on the criteria described below:

**True positive:** The diagnosis output by the NLP program is considered true positive when the disease status is consistent with the clinician-recorded diagnosis documented in the periodontal evaluation form.

Example: The clinician-recorded diagnosis stated, **“mild chronic generalized periodontitis.”** If the natural language processing algorithm has extracted this diagnosis as 1) **disease type=periodontitis**, 2) **severity=mild**, 3) **extent=generalized**, and **onset=chronic**, then this diagnosis is assigned as “true positive” class (See Table 3).

**SM Table 3:** An example of a true positive case output of the NLP program for a clinician- recorded diagnosis of “**mild chronic generalized periodontitis”**

| NLP Diagnosis | Class | Severity | Onset | Class | Extent | Class |
| --- | --- | --- | --- | --- | --- | --- |
| Periodontitis | TP | mild | chronic | TP | generalized | TP |

**False positive:** The NLP algorithm’s output for diagnosis is considered false positive when the output indicates a positive disease status even though the patient record indicates no disease. For example, if the NLP algorithm determines a patient to have “gingivitis or periodontitis,” although the clinician-recorded diagnosis indicates “no disease/healthy” status, then it’s a false positive case (See examples in Tables 4 and 5).

**SM Table 4:** Example of a false positive case output by the NLP program for a clinician-recorded diagnosis of “mild generalized gingivitis”

| NLP Diagnosis | Class | Severity | Onset | Class | Extent | Class |
| --- | --- | --- | --- | --- | --- | --- |
| Periodontitis | FP | Mild | Chronic | FP | Generalized | TP |

**SM Table 5:** Examples of false positive case outputs by the NLP program

| Clinician recorded diagnosis | NLP Algorithm’s Output | False Positives |
| --- | --- | --- |
| Disease Status | | |
| Healthy | Gingivitis | False Positive |
| Healthy | Periodontitis | False Positive |
| Gingivitis | Periodontitis | False Positive |
| Severity | | |
| Mild | Moderate | False Positive |
| Mild | Severe | False Positive |
| Moderate | Severe | False Positive |
| Extent | | |
| Localized | Generalized | False Positive |

**False negative:** The NLP program’s output is considered false negative when it classifies a patient’s disease status as ‘no disease or healthy’ although the patient has gingivitis or periodontitis. For example, if NLP algorithm classifies a patient who have a “gingivitis or periodontitis” diagnosis in the clinical notes as “no disease/healthy” then it’s considered false negative case (See example Tables 11, 12 below).

**SM Table 6:** Example of a false negative output for a clinician-recorded diagnosis of “mild chronic generalized gingivitis”

| NLP Diagnosis | Class | Severity | Onset | Class | Extent | Class |
| --- | --- | --- | --- | --- | --- | --- |
| No disease | FN | mild | chronic | TP | Localized | FN |

**SM Table 7:** Possible examples to correctly identify false negative cases

| Actual disease status (clinician recorded diagnosis) | NLP Algorithm’s Output | | False Negative |
| --- | --- | --- | --- |
| Disease Status | | | |
| Gingivitis | Healthy | | False Negative |
| Periodontitis | Healthy | | False Negative |
| Severity | | | |
| Moderate | Mild | | False Negative |
| Severe | Mild | | False Negative |
| Severe | Moderate | | False Negative |
| Extension | | | |
| Generalized | Localized | | False Negative |
| **SM Table 8:** Demographics of patients who received at least one comprehensive oral evaluation at Indiana University School of Dentistry between January 1, 2009, and December 31, 2014 | | | |
| Patient characteristics | | Number of patients (N) (%) | |
| Age | | | |
| 18-29 | | 6,492 (22) | |
| 30-44 | | 7,233 (25) | |
| 45-64 | | 10,907 (38) | |
| 65 and more | | 4,276 (15) | |
| Total | | 28,908 (100) | |
| Gender | | | |
| Female | | 15,572 (54) | |
| Male | | 13,242 (46) | |
| Transgender | | 8 (0) | |
| Other | | 86 (0.2) | |
| Total | | 28,908 (100) | |
| Race/Ethnicity | | | |
| Caucasian | | 14,038 (49) | |
| African American | | 3,856 (13) | |
| Hispanic | | 1,902 (7) | |
| Asian | | 580 (2) | |
| Other | | 164 (1) | |
| Multiracial | | 41 (0) | |
| American Indian | | 14 (0) | |
| Pacific Islander | | 2 (0) | |
| Unknown | | 31 (0) | |
| Missing | | 8,280 (29) | |
| Total | | 28,908 (100) | |
| Insurance status | | | |
| Self-pay | | 13, 067 (45) | |
| Private insurance | | 12, 751 (44) | |
| Government insurance | | 3,090 (11) | |
| Total | | 28,908 (100) | |
